# Majorbio Cloud 2024: Update single‐cell and multiomics workflows

**DOI:** 10.1002/imt2.217

**Published:** 2024-06-25

**Authors:** Chang Han, Caiping Shi, Linmeng Liu, Jichen Han, Qianqian Yang, Yan Wang, Xiaodan Li, Wenyao Fu, Hao Gao, Huasheng Huang, Xianglin Zhang, Kegang Yu

**Affiliations:** ^1^ Shanghai Majorbio Bio‐Pharm Technology Co. Ltd. Shanghai China

## Abstract

Majorbio Cloud (https://cloud.majorbio.com/) is a one‐stop online analytic platform aiming at promoting the development of bioinformatics services, narrowing the gap between wet and dry experiments, and accelerating the discoveries for the life sciences community. In 2024, three single‐omics workflows, two multiomics workflows, and extensions were newly released to facilitate omics data mining and interpretation.

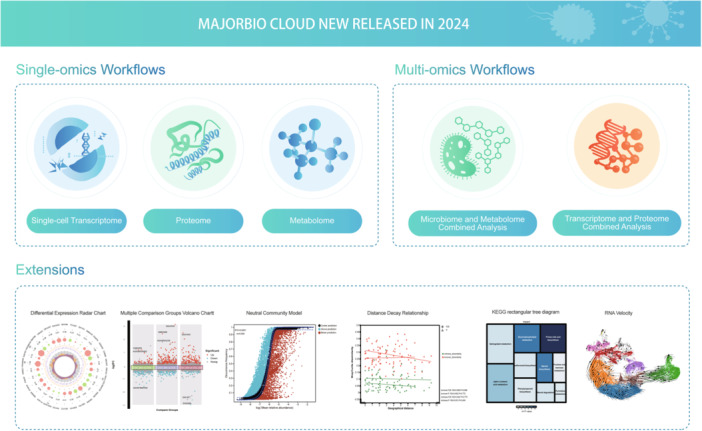

Advances in high‐throughput multiomics technologies have significantly influenced life science and basic medical research, specifically based on multiomics data, including genomic/transcriptomic sequencings and proteomic/metabolomic mass spectra, paving the way for the discovery of novel predictive biomarkers for predicting treatment response from diverse dimension levels. The state‐of‐the‐art multiomics technologies have enabled researchers to understand biological processes and molecular functions in health and disease. The emerging novel omics strategies and instruments continue to evolve toward higher throughput and lower detection costs. The evergrowing quantity of multiomics data needed to have access to the resources and be analyzed in an easy, fast, and accurate way. The requirement for the development and application of appropriate bioinformatic tools and pipelines to interpret these data is urgent. Two key elements of omics are automatic data analysis and data visualization. Bioinformatics analysis platforms, such as Cell Ranger [1], MetaboAnalyst [2], GEPIA2 [3], and iNAP [4] provide web interfaces to access the data and computational results. However, these interaction‐friendly web services are designed for a single type of omics.

Majorbio Cloud (https://cloud.majorbio.com/) offers an easy and powerful approach to profiling the bulk transcriptome, single‐cell transcriptome, proteome, metabolome, metagenome, and other omics data. It facilitates researchers to analyze complex multiomics data and infer the biological meaning of integrated omics data. Since Majorbio Cloud's first publication in iMeta, it has attracted the attention of researchers around the world and has been widely used by researchers who are not specialists in omics or bioinformatics [5]. Furthermore, it is an interactive communication and omics knowledge dispersion platform.

## WORKFLOW 1: SINGLE‐CELL TRANSCRIPTOMICS WORKFLOW

**Figure 1:**
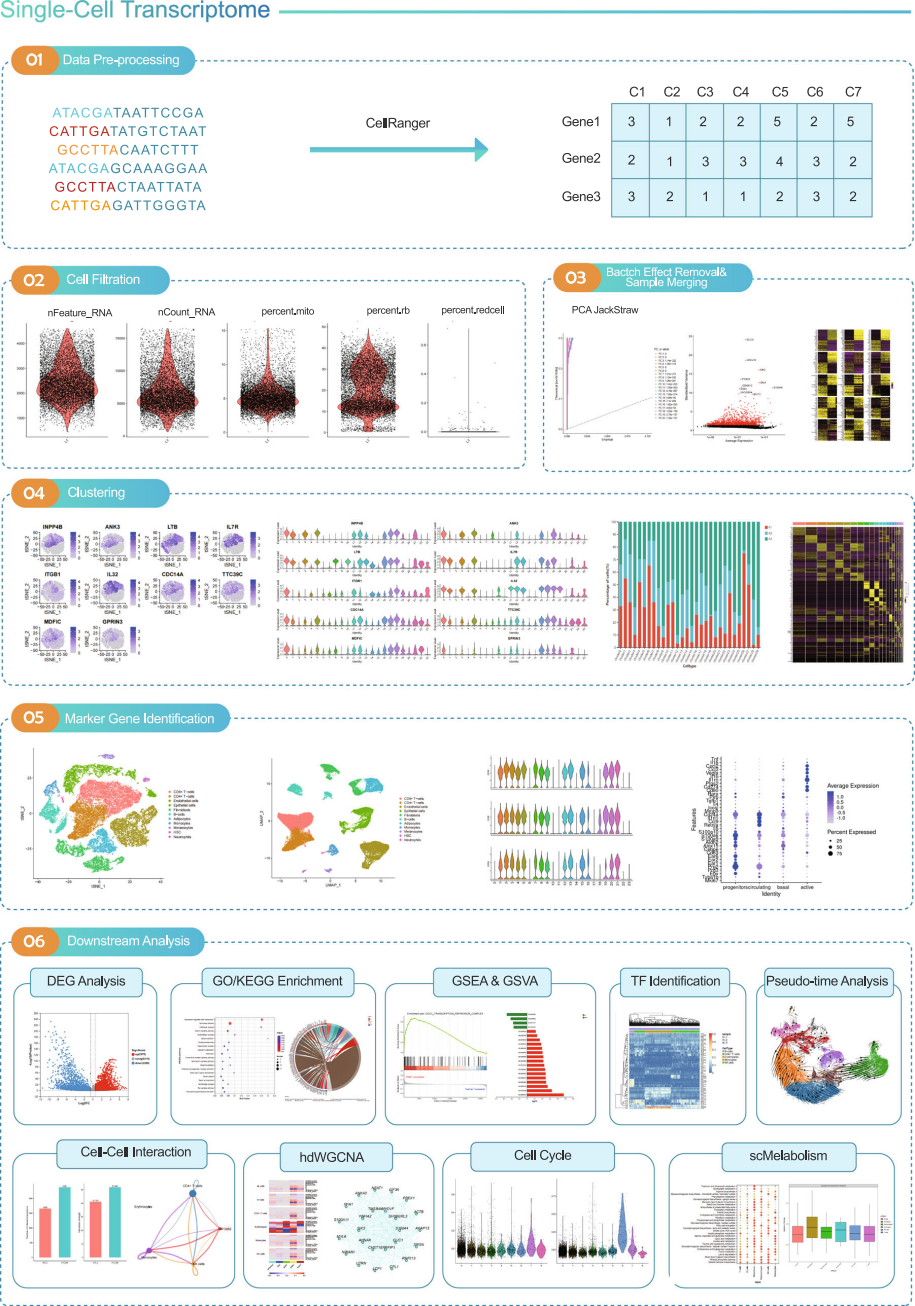
Single‐cell transcriptomics workflow. The workflow includes six steps: (1) data preprocessing, (2) cell filtration, (3) batch effect removal and sample merging, (4) clustering, (5) marker gene identification, and (6) downstream analysis. DEG, differential expression gene; GSEA, gene set enrichment analysis; TF, transcription factor.

Single‐cell RNA sequencing is an emerging technology for high‐throughput sequencing analysis of genetic material at the level of individual cells [6]. It has been widely applied in immunology, developmental biology, oncology, cardiology, and neurobiology. The single‐cell transcriptomics workflow is an easy‐to‐use and effective pipeline for high‐dimensional single‐cell transcriptome data mining, including the following six steps: (1) data preprocessing; (2) cell filtration; (3) batch effect removal and sample merging; (4) clustering; (5) marker gene identification; and (6) downstream analysis. The detailed process is as follows: Reads are processed using the Cell Ranger (v7.1.0) with default parameters. FASTQ files generated by the Illumina sequencer are aligned to the genome. The Seurat package was used for cell normalization and regression based on the unique molecular identifier counts for each sample and mito % to obtain the scaled data, which was normalized by the function NormalizeData for further analysis. The function FindVariableGenes was used to calculate highly variable genes across the single cells. Unsupervised cell cluster results were generated based on the principal component analysis's (PCA's) top 30 principal components by applying the graph‐based cluster method (resolution 0.8) in the Seurat package. For subclustering, we applied the same procedure of scaling, dimensionality reduction, and clustering to a specific set of data (usually restricted to one cell type). For each cluster, we used the Wilcoxon Rank‐Sum test to find significant deferentially expressed genes comparing the remaining clusters. SingleR [7] and known marker genes were used to identify cell types. Downstream analysis, such as differential expression genes and pathway enrichment of different cell types, pseudo‐time analysis, and cell communication analysis, could be used to reveal the functions, states, and interactions of various types of cells in a sample (Figure 1).

## WORKFLOW 2: PROTEOMICS WORKFLOW

**Figure 2:**
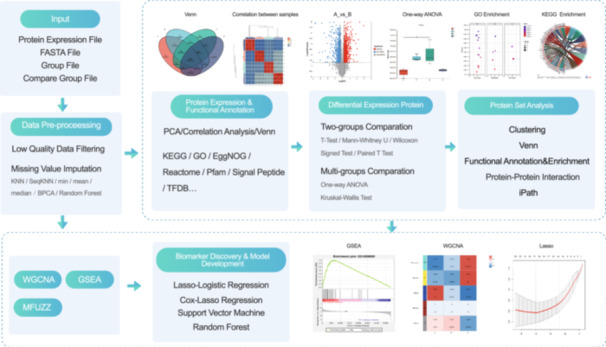
Proteomics workflow. The standard proteomics workflow consists of seven main modules. Biomarker discovery and model development modules are additionally available for medical cohort research. ANOVA, analysis of variance; BPCA, bayesian PCA; GO, gene ontology; FASTA, a text‐based format for representing either nucleotide sequences or amino acid (protein) sequences, in which nucleotides or amino acids are represented using single‐letter codes; GSEA, gene set enrichment analysis; KEGG, kyoto encyclopedia of genes and genomes; KNN, K‐nearest neighbor classification; PCA, principal component analysis; TFDB, transcription factor database; WGCNA, weighted gene correlation network analysis.

The proteomics workflow is a user‐friendly, comprehensive pipeline for data‐independent acquisition mass spectrometry‐based, label‐free quantitation (LFQ), and Tandem mass tag‐based quantitative proteomics data processing, analysis, and interpretation (Figure 2). The standard proteomics workflow consists of seven main modules: data processing, protein expression and functional annotation, statistical analysis, protein set analysis, weighted gene correlation network analysis (WGCNA), gene set enrichment analysis (GSEA), and time‐series data analysis. An additional module, biomarker discovery and model development is provided for medical cohort research.

The function of the proteomics data processing module is low‐quality data filtering and missing value estimation. The protein expression and functional annotation module includes Venn, PCA, correlation analysis, and functional annotations based on databases or software. Paired/unpaired *t* test, analysis of variance, Kruskal–Wallis test, and post‐hoc test are provided for statistically significant differences in protein identification. A protein set is a protein list that is related to the phenotype of the research object according to the protein expression profile, functional annotation, biological pathway enrichment, and research background. Users can generate protein sets of their interest and interpret the data via clustering, protein–protein interaction, pathway analysis, functional enrichment, and so on. LASSO‐Logistic/Cox regression, Random Forest [8], and SVM [9] can be used for disease risk prediction, early diagnosis, prognosis monitoring, and response to treatment.

## WORKFLOW 3: METABOLOMICS WORKFLOW

Metabolomics research is primarily based on the use of liquid/gas chromatography‐mass spectrometry and nuclear magnetic resonance spectroscopy to detect, identify, and quantify small molecule metabolites in organisms [10]. Metabolomics data is large and complex, often requiring specialized data analysis software as well as extensive knowledge of cheminformatics, bioinformatics, and statistics. To enable users to perform metabolomics data analysis easily and quickly, we provide a comprehensive solution for metabolomics workflow (Figure S1).

The standard metabolomics workflow consists of five steps: (1) Data preprocessing: the methods mainly include filtering the missing values of the original data, missing value estimation, data normalization, quality control verification, and data transformation. (2) Sample comparison analysis: multivariate statistical analysis was performed by PCA and partial least squares discriminant analysis (PLS‐DA); (3) metabolite annotation: metabolites were annotated in kyoto encyclopedia of genes and genomes (http://www.genome.jp/kegg/) and human metabolome database (https://hmdb.ca/) databases; (4) differential expression metabolites analysis: a combination of multidimensional analysis and single‐dimensional analysis was used to screen differential metabolites between groups; and (5) metabolite set analysis: analysis and visualization of the key or differential expression metabolites, such as metabolite clustering, correlation analysis, and so on. Moreover, we also provide some advanced analyses to reveal the mysteries of biological processes, such as biomarker discovery by random forest, support vector machine (SVM), and so on.

## THE INTEGRATED MULTIOMICS PIPELINES

The multiomics technologies facilitate researchers to uncover underlying mechanistic insights into disease pathophysiology and delineate the landscape of clinical phenotypes. Multiomics provides an integrated perspective across multiple levels, while single omics data can only partially explain one aspect of complex biological processes [11]. The transcriptomic and proteomic data combined analysis pipeline supports differential expression analysis, correlation between messenger RNA (mRNA) and protein abundance, functional annotation and enrichment, GSVA [12], and interactive visualization including Venn, quadrant diagram, nine quadrant diagram, bubble plot, box plot, and donut plot. The pipeline enables a combined, complementary insight, which improves a comprehensive understanding of biological molecular processes from mRNA to protein.

The microbiome and metabolome association analysis workflow can be used to analyze the association between species/function and metabolites so as to help establish the logical association between “species/function—metabolite—phenotype/target organ.” The results systematically delineate the regulatory mechanisms of biological processes of different dimensions. To facilitate the intuitive presentation of scientific findings, the workflow provides a broad diversity of analyses. The main analysis contents are as follows: (1) annotation and abundance (species, KEGG orthology genes, and metabolic species) of the single‐omics feature set; (2) procrustes and orthogonal partial least squares discriminant analysis (O2PLS) are used to analyze the synergy between microbial communities and metabolites and to screen the species and metabolites that contributed the most to distinguishing different groups of samples. (3) To explain the association between key flora and metabolites can be achieved by HCLUST correlation analysis, Mantel test network heatmap, expression correlation heatmap and chord map, expression correlation network, linear regression analysis, MaAsLin analysis, and canonical correlation analysis. (4) The microbiome and metabolome data are used to form a combinatorial marker panel, and four integrated machine learning algorithms, including random forest, SVM, least absolute shrinkage and selection operator (LASSO), and logistic regression, are used to efficiently screen predictive biomarkers. In addition, A metabolic network‐based tool for inferring mechanism‐supported relationships in microbiome‐metabolome data (MIMOSA2) [13], mmvec [14], and WGCNA are available for further analyses to interpret the possible interactions between microorganisms and metabolites. (5) Metabolite detection technology is used to detect intermediate metabolites. In combination with the metabolic pathways predicted by metagenome data, the downstream metabolic pathways can be reconstructed to obtain a complete microbial metabolic pathway.

## “PIPELINE + EXTENSIONS,” A NEW INTERACTIVE ANALYSIS MODE

To improve the user experience and expand the depth of analysis, we have developed a completely new interactive analysis mode. Taking the eukaryotic reference transcriptome analysis pipeline as an example, users can select the data table generated in the pipeline and set parameters in extension tools to complete more in‐depth data mining. The intermediate data generated by the workflow is extracted into javascript object notation format parameters, encrypted transmission to a specific tool via base64, and parameter parsing is finished in the tool (Figure S2). Twenty‐eight eukaryotic reference transcriptome analysis pipeline‐specific extension tools are available for users, including integrative genomics viewer visualization, multiref‐genome blast, differential expression genes radar chart, hyperbolic curve volcano chart, circos chart, single gene GSEA, multipathways GSEA, and so on.

## USERS AND PUBLICATIONS

Since October 2016, more than 150,000 scientific and clinical research users, involving over 9000 well‐known universities and institutions, have completed more than 600,000 omics data mining tasks on the Majorbio Cloud platform. In 2024, 2015 journal articles cited Majorbio Cloud in their methods. 20, 62, and 393 research articles have been published with the facilitation of the single‐cell transcriptomics workflow, proteomics workflow, and metabolomics workflow for data mining, respectively. We will constantly update and iterate the platform to make our users delve more deeply into the omics data.

## AUTHOR CONTRIBUTIONS

Jichen Han, Chang Han, Caiping Shi, and Linmeng Liu conceived the platform and idea. Linmeng Liu, Caiping Shi, and Wenyao Fu implemented the MIST main code. Chang Han, Qianqian Yang, Yan Wang, and Xiaodan Li designed the graphical user interface. Chang Han, Yan Wang, Xiaodan Li, and Qianqian Yang wrote the manuscript. Chang Han was responsible for editing and revising the manuscript. All authors contributed to the development of Majorbio Cloud. All authors have read the final manuscript and approved it for publication.

## CONFLICT OF INTEREST STATEMENT

The authors declare no conflict of interest.

## Supporting information


**Figure S1:** Metabolomics workflow.
**Figure S2:** “Pipeline + Extensions” interactive analysis mode.

## Data Availability

Supporting Information (graphical abstract, Supporting Information Table) may be found in the online DOI or iMeta Science http://www.imeta.science/.
