# Supplementary material for: Majorbio Cloud 2024: Update single‐cell and multiomics workflows
Source: Imeta. 2024 Jun 25;3(4):e217. doi: 10.1002/imt2.217 (PMC11316920; doi:10.1002/imt2.217)
Supplement: Supplementary file 1 — Figure S1: Metabolomics workflow. Figure S2: “Pipeline + Extensions” interactive analysis mode. [file IMT2-3-e217-s001.docx]

**Supporting information to**

**Majorbio Cloud 2024: update single-cell and multi-omics workflow**

**Running title: Majorbio Cloud 2024 Update**

Chang Han^1#*^, Caiping Shi^1#^, Linmeng Liu^1#^, Jichen Han^1#*^, Qianqian Yang^1^, Yan Wang^1^, Xiaodan Li^1^, Wenyao Fu^1^, Hao Gao^1^, Huasheng Huang^1^, Xianglin Zhang^1^, Kegang Yu^1^

^1^Shanghai Majorbio Bio-Pharm Technology Co., Ltd, Shanghai 201318, China.

^#^These authors contribute equally: Chang Han, Caiping Shi, Linmeng Liu, Jichen Han

*Correspondence: jichen.han@majorbio.com (Jichen Han); [chang.han@majorbio.com](mailto:chang.han@majorbio.com) (Chang Han)


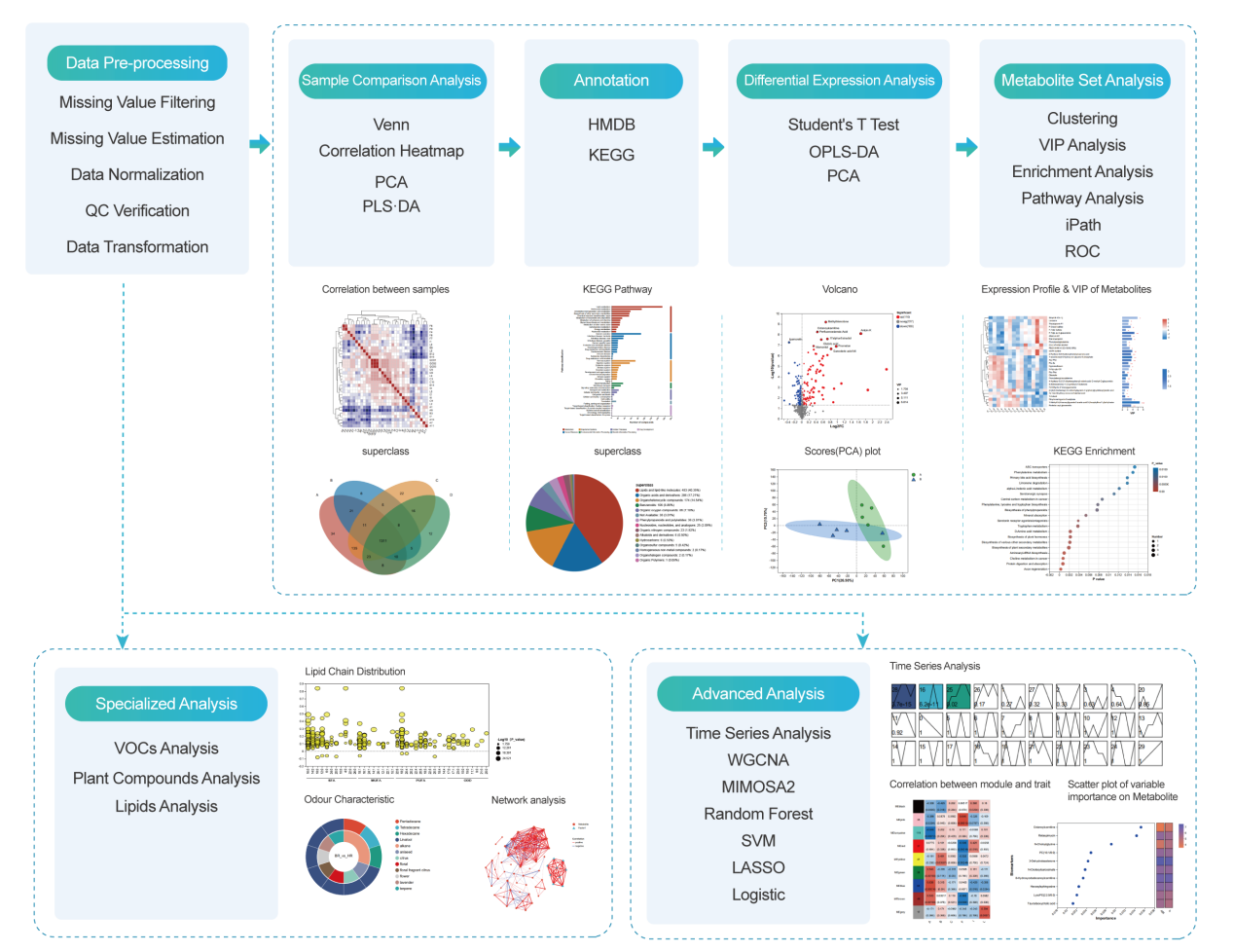


**Figure S1 Metabolomics workflow.** The standard metabolite analysis workflow consists of 5 steps: (1) Data pre-processing; (2) Sample comparison analysis; (3) Metabolite annotation; (4) Differential expression metabolites analysis; (5) Metabolite set analysis. We offer an additional module for customers who specialize in VOCs, plant compounds and lipids research. Advanced analyses facilitate biomarker discovery. QC: quality control; ROC: Receiver Operating Characteristic Curve; VOCs: volatile organic compounds; SVM: Support Vector Machine.


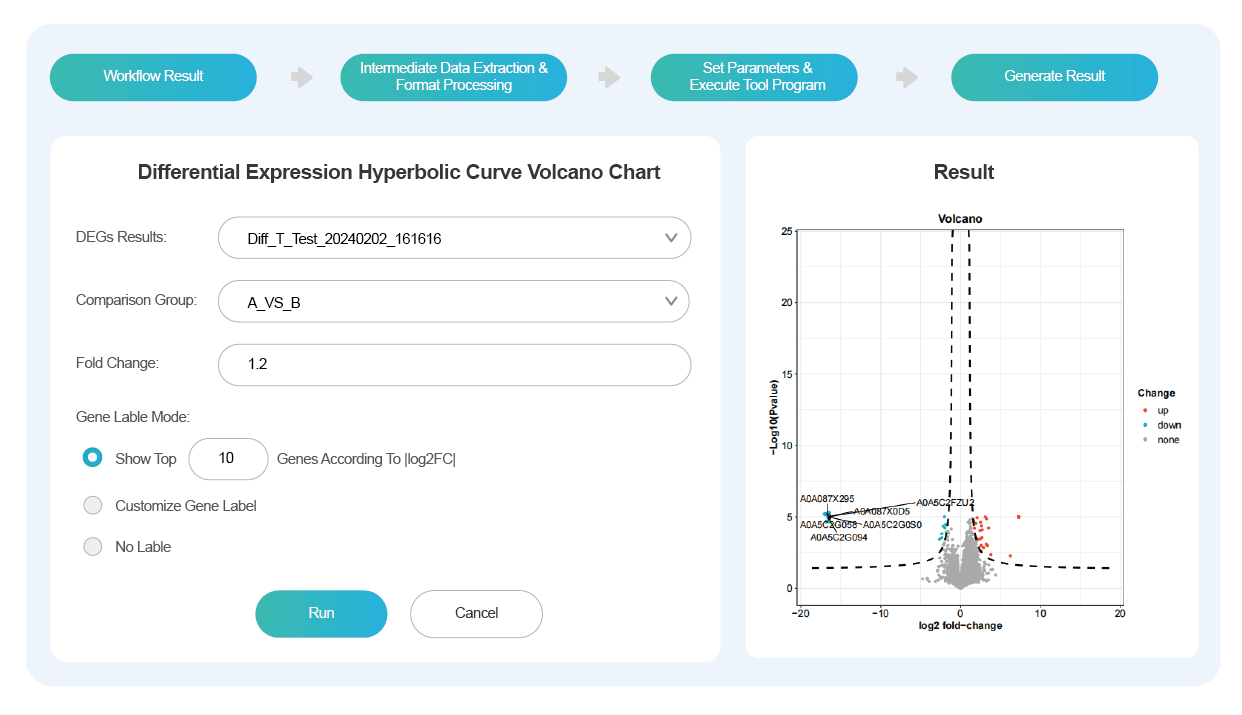
**Figure S2 "Pipeline + Extensions" interactive analysis mode.** The intermediate data of a workflow can be analyzed using extension tools. Web page and visualization result of differential expression hyperbolic curve volcano chart.
